# A novel nomogram with preferable capability in predicting the overall survival of patients after radical esophageal cancer resection based on accessible clinical indicators: A comparison with AJCC staging

**DOI:** 10.1002/cam4.3878

**Published:** 2021-06-15

**Authors:** Xinye Li, Jinming Xu, Linhai Zhu, Sijia Yang, Li Yu, Wang Lv, Jian Hu

**Affiliations:** ^1^ Department of Thoracic Surgery The First Affiliated Hospital School of Medicine Zhejiang University Hangzhou China

**Keywords:** AJCC, esophageal cancer, nomogram, overall survival, radical resection, risk classification

## Abstract

**Background:**

Esophageal cancer (EC) is a malignant tumor with high mortality. Nomogram is an important tool used in clinical prognostic assessment. We aimed to establish a novel nomogram to predict the overall survival (OS) of EC patients after radical esophagectomy.

**Methods:**

Data pertaining to the survival, demography, and clinicopathology of 311 EC patients who underwent radical esophagectomy were retrospectively investigated. The nomogram was established based on Cox hazard regression analysis. The calibration curves and Harrell's concordance index (C‐index) were used to verify the predictive accuracy and ROC curves were used to assess the efficacy of the nomogram. Kaplan–Meier curves showed the prognostic value of the related risk classification system. Pearson correlation test was performed to determine the correlation between the risk classification system and TNM staging.

**Results:**

The median OS and 5‐year survival rates in the primary and validation cohorts were 44 months and 29.8%, and 52 months and 27.1%, respectively. We used six independent prognostic factors—age, Sex, AGR, PRL, N stage, and PNI—in the nomogram. The C‐index of nomogram was 0.75 and 0.70 in the primary and validation cohorts, respectively. Calibration curves indicated high consistency between actual and predicted OS. ROC curves showed that nomogram has a better efficacy compared with TNM staging in both cohorts. Patients were divided into three risk groups according to the total nomogram score, the median OS in each group was significantly different in both cohorts. Furthermore, the risk classification system was strongly correlated with the T and N staging system and exhibited a better OS prediction capability.

**Conclusions:**

We established a novel and practical nomogram with a subordinate risk classification system to predict the OS of patients after radical esophagectomy. Compared with AJCC staging, this nomogram had preferable clinical capability in terms of individual prognosis assessment.

## Graphical abstract

We built and validated a novel nomogram to predict OS in radical esophageal cancer resection patients, the nomogram showed considerable prognostic capability especially compared with AJCC staging. Furthermore, a related risk classification system was generated for clinical evaluation.

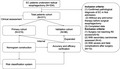

## 1 INTRODUCTION

Esophageal cancer (EC), a serious threat to human life and health, has increasing morbidity and mortality rate worldwide.^1^ Additionally, as a result of unhealthy diet and living habits, EC ranks as the 6th most common malignancy, with 4th highest mortality rate in China.^2^ In China, the most common pathological type of EC is squamous cell carcinoma, while in western nations, it is adenocarcinoma. Radical resection is the major therapy for EC. However, due to the lack of accurate early diagnosis approaches and effective prognostic indicators, the 5‐year overall survival (OS) rate of EC is around 30%.^3^ Recently, with the evolution of neoadjuvant chemoradiotherapy, the prognosis of radical EC surgery has markedly improved.^4^ An accurate prognostic evaluation instrument that contributes in formulating more precise and individualized prognostic assessment is of great significance to extend the life of patients with EC.

Various factors have a crucial influence on the prognosis of cancer‐related patients, are regarded as the prognostic factors. With decades of widespread multicenter clinical research, the tumor–node–metastasis (TNM) staging system by the American Joint Committee on Cancer (AJCC), as well as age, pathological type, and treatment patterns, are approved as the conventional independent prognostic indicators for EC.^5^ However, because of the confounding factors that affect the prognosis of EC, single‐handed AJCC staging cannot predict the prognosis satisfactorily, especially for patients with similar staging.^6^, ^7^

Nomograms are widely utilized in cancer prognosis because of their ability to transform the statistical prediction results into a comprehensive quantitative and visual estimate of the probability of an event, such as death or recurrence.^8^, ^9^ Previous studies using nomograms to assess the outcomes in patients with EC indicated that nomograms have higher prognostic assessment ability and accuracy compared with the traditional prognostic methods, such as AJCC staging, pathological grading, and other single prognostic indicator, even in a subgroup analysis.^10^, ^11^ The establishment of nomograms integrating conventional factors for EC has been noted in few studies.^12^, ^13^, ^14^ Furthermore, a series of indicators that can be converted by routine clinical examination parameters showed considerable prognostic potential in patients with EC, such as albumin‐to‐globulin ratio (AGR), neutrophil‐to‐lymphocyte ratio (NLR), platelet‐to‐lymphocyte ratio (PLR), prognostic nutritional index (PNI), and low‐density lipoprotein (LDL).^15^, ^16^, ^17^, ^18^ However, only few studies on nomogram combining these certified novel prognostic factors for EC patients have been published.

In this study, we aimed to establish and verify a nomogram integrating clinically available prognostic factors for OS prediction in EC patients who underwent radical esophagectomy and compare its prediction efficacy to AJCC staging. The nomogram will be a novel, comprehensive, and clinically convenient prognostic tool, which provides new insights regarding patient consultation, prognosis assessment, and formulating follow‐up strategy.

## 2 MATERIALS AND METHODS

### 2.1 Patients

Data pertaining to the survival, demography, and clinicopathology of 311 patients who underwent radical resection of EC between January 2008 and December 2013 in The First Affiliated Hospital of Zhejiang University School of Medicine were retrospectively collected and analyzed. The inclusion criteria were as follows: (i) patients with confirmed pathological diagnosis of EC (squamous or adenous) at first admission; (ii) patients who did not receive any antitumor therapy before surgical esophagectomy; and (iii) patients for whom all relevant data were available. The exclusion criteria were as follows: (i) surgery not reaching R0 excision; (ii) patients with secondary carcinomas assessed by clinical history, imaging‐based examination, or routine laboratory tests; and (iii) patients with severe and fatal complications after surgery. This study was conducted in accordance with the principles of the Declaration of Helsinki and the study protocol was approved by the Clinical Research Ethics Committee of The First Affiliated Hospital of Zhejiang University School of Medicine.^19^

We formulated postoperative treatment strategy according to the National Comprehensive Cancer Network (NCCN) guidelines and individual feature of each patients. Postoperative treatments include radiotherapy, chemotherapy, concurrent radiotherapy, and chemotherapy, all radical esophagectomy patients will be required to formulate postoperative treatment regimen under multidisciplinary discussions, especially those with local advanced stage.^20^

The variables of each patient included demographic data: age, sex, smoking and alcohol status, and BMI; clinicopathological data: carcinoembryonic antigen (CEA, ng/mL), tumor location, pathological type, differentiated degree, T and N stages, and low‐density lipoprotein (LDL, mmol/L); and available prognostic indicator: AGR, NLR, PLR, PRL, and PNI. Age, sex, smoking and alcohol status, and BMI were obtained from the hospitalization information and medical history, CEA, NLR, PLR, LDL, and PNI were tested and calculated within 1 week before surgery, AJCC T and N stages, pathological type, tumor location, and PRL were obtained after surgery within 2 weeks. The description and calculation formula of related indicators are as follows:

A. BMI (Body mass index) = body weight (kg)/height (m^2^).

B. T and N stages: according to the 7th edition AJCC TNM staging guideline of EC.

C. AGR: Albumin‐to‐globulin ratio.

D. NLR: Neutrophil‐to‐lymphocyte ratio.

E. PLR: Platelet‐to‐lymphocyte ratio.

F. PRL: Positive lymph nodes ratio.

G. LDL: Low‐density lipoprotein.

H. PNI: Prognostic nutritional index, calculated as 10 × serum albumin (g/dL) + 0.005 × lymphocyte count (per mm^3^).^21^

### 2.2 Study design and outcome definition

**FIGURE 1:**
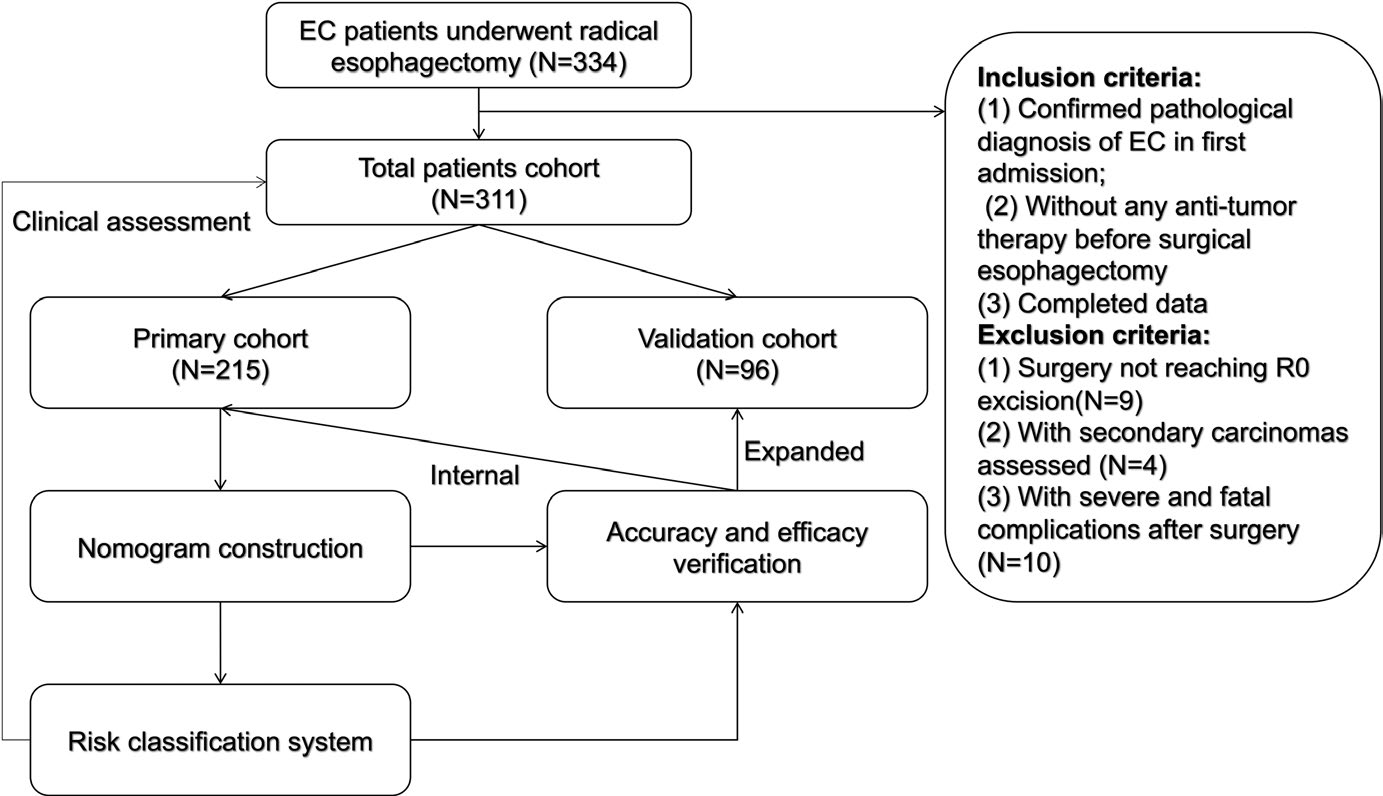
A flow chart for the design of our study. A total of 311 EC patients underwent radical esophagectomy were randomly divided into primary and validation cohorts. A nomogram was built based on the primary cohort, the accuracy and efficacy of the nomogram and relevant risk classification system were verified in both primary and validation cohorts. Finally, the clinical assessment of risk classification system was performed in total patients. EC, Esophageal cancer

All included patients were randomly allocated into primary cohort (215 of 311, 69.1%) and validation cohort (96 of 312, 30.9%). The patients were followed‐up via clinic visits or telephone interviews, and the OS of the patients was recorded for the principal outcome. The follow‐up data of each patient were updated every 3 months during the first 2 years, every 6 months in the next 2 years, and annually thereafter until December 2018. The OS was calculated from the time of radical esophagectomy to the time of death or until the last follow‐up. The study design is described in a flow chart in Figure 1.

### 2.3 Statistical methods

Categorical variables were classified based on clinical findings, while continuous variables were transformed into categorical variables based on cut‐off points determined by median values or recognized clinical findings. In the balance control between the primary and validation cohorts, chi‐square test was used to show the difference in proportion in each variable.

In the primary cohort, we performed univariate and multivariate Cox proportional hazards regressions to assess OS, and variables with *P* values less than 0.05 in multivariable Cox proportional hazards regression were used to establish a predictive nomogram model.^8^

The predictive accuracy of the nomogram model was performed using Harrell's Concordance Index (C‐index) and calibration curves. A larger C‐index value indicated more efficient ability to correctly discriminate the prediction of outcome.^22^ Calibration curves for 1‐year, 3‐year, and 5‐year OS were made using a bootstrap method to quantify the modeling strategy of the nomogram. Receiving operative characteristics (ROC) curves were used to compare the prediction efficacy of nomogram with AJCC staging. All the methods above were equally implemented in validation cohorts for expanded verification.

Furthermore, based on the total score from the predictive nomogram model, patients were divided into three risk groups (low risk, intermediate risk, and high risk) in both the primary and validation cohorts. Kaplan–Meier curves and log‐rank test were used to illustrate and compare the OS of patients in the different risk groups.

In the total cohort, chi‐square and Pearson correlation tests were applied to show the correlation between the risk classification system and AJCC TNM staging system.

All statistical analyses and graphics were performed using the SPSS 25.0 statistical package (SPSS Inc., Chicago, IL, USA), R version 3.6.1 (R Foundation for Statistical Computing, Vienna, Austria), and GraphPad Prism 8.2.1 (GraphPad Software, La Jolla, CA). *P* values less than 0.05 in two‐tailed test were considered statistically significant.

## 3 RESULTS

### 3.1 Characteristics of patients in randomized primary and validation cohorts

A total of 311 eligible patients who underwent radical esophagectomy were enrolled and randomly grouped into primary and validation cohorts. The median age of all patients was 63 years (range, 40–83; SD = 7.76). Moreover, the median OS and 5‐year survival rate were 44 months and 29.8%, and 52 months and 27.1% in the primary and validation cohorts, respectively. Continuous variables, including BMI, NLR, PLR, AGR, LDL, CEA, PNI, and PRL, were transformed into binary categorical variables according to cut‐off values determined by median or clinically recognized results. All patients’ baseline demographic and clinicopathological characteristics were balanced between the primary and validation cohorts (Table 1).

**TABLE 1:** Baseline demographic and clinicopathological characteristics in primary and validation cohorts

Characteristics	Primary cohort (N=215), no. (%)	Validation cohort (N=96), no. (%)	*P* value^a^
Age (years)			
≥65	87 (40.5)	36 (37.5)	0.621
<65	128 (59.5)	60 (62.5)	
Sex			
Male	190 (88.4)	82 (87.5)	0.467
Female	25 (11.6)	14 (12.5)	
Smoke status			
Yes	144 (67.0)	69 (71.9)	0.390
No	71 (33.0)	27 (28.1)	
Alcohol status			
Yes	136 (63.3)	62 (64.6)	0.822
No	79 (36.7)	34 (35.4)	
BMI			
≥22.13	116 (54.0)	59 (61.5)	0.218
<22.13	99 (46.0)	37 (38.5)	
NLR			
≥2.22	106 (49.3)	48 (50)	0.909
<2.22	109 (50.7)	48 (50)	
PLR			
≥123.51	105 (48.8)	49 (51.0)	0.719
<123.51	110 (51.2)	47 (49.0)	
AGR			
≥1.61	112 (52.1)	44 (45.8)	0.308
<1.61	103 (47.9)	52 (54.2)	
LDL (mmol/L)			
≥2.42	111 (51.6)	50 (52.1)	0.941
<2.42	104 (48.4)	46 (47.9)	
CEA (ng/mL)			
≥5	27 (12.6)	13 (13.5)	0.811
<5	188 (87.4)	83 (86.5)	
PNI			
≥50	109 (50.7)	45 (46.9)	0.533
<50	106 (49.3)	51 (53.1)	
Tumor location			
Upper	14 (7.0)	5 (5.2)	0.658
Middle or lower	201 (93.0)	91 (94.8)	
Differentiation			
Poor	24 (11.2)	7 (7.3)	0.182
Moderate	119 (55.3)	47 (49.0)	
Well	72 (33.5)	42 (43.7)	
Pathological type			
Squamous	213(99.1)	95(99.0)	0.926^c^
Adenocarcinoma	2(0.9)	1(1.0)	
T stage^b^			
Tis/I	39 (18.1)	16 (16.7)	0.463
II	36 (16.7)	10 (10.4)	
III	122 (56.7)	62 (64.6)	
IV	18 (8.4)	8 (8.3)	
N stage^b^			
N0+N1	176 (81.9)	78 (81.3)	0.898
N2+N3	39 (18.1)	18 (18.7)	
PRL			
≥0.1	68 (31.6)	29 (30.2)	0.803
<0.1	147 (68.4)	67 (69.8)	

Abbreviations: AGR, Albumin‐to‐globulin ratio; BMI, Body mass index; LDL, Low‐density lipoprotein; NLR, Neutrophil‐to‐lymphocyte ratio; PLR, Platelet‐to‐lymphocyte ratio; PNI, Prognostic Nutritional Index; PRL, Positive lymph nodes ratio.

^a^ Chi‐square test.

^b^ T and modified N staging based on 7th AJC.

^c^ Fisher exact test.

### 3.2 Cox regression analysis and optimization of variables for nomogram in the primary cohort

Univariable and multivariable Cox hazard regression analyses for OS in primary cohort are shown in Table 2. In univariate Cox hazard regression model, variables, including age (≥65 years vs <65 years), sex (male vs female), differentiation (poor vs moderate vs well), AJCC T stage, modified N stage (N0–N1 vs N2–N3), PLR (≥123.51 vs <123.51), NLR (≥2.22 vs <2.21), AGR (≥1.61 vs <1.61), CEA (≥5 ng/mL vs <5 ng/mL), PRL (≥0.1 vs <0.1), and PNI (≥50 vs <50), were significantly related to OS. For the optimization of variables to establish a nomogram predictive model, a subsequent multivariable Cox hazard regression analysis of the variables above was done, and six independent factors (age, sex, AGR, N stage, PNI, and PRL) were chosen for nomogram validation.

**TABLE 2:** Univariable and multivariable Cox hazard analysis for OS in primary cohort

Univariable analysis	HR	95% CI of HR	*P* value^a^
Age (years)			
≥65 vs <65	1.568	1.095–2.244	**0.014**
Sex			
Male vs female	2.889	1.346–6.201	**0.006**
Smoke status			
Yes vs No	1.263	0.855–1.866	0.240
Alcohol status			
Yes vs No	0.855	0.614–1.275	0.511
Tumor location			
Middle/lower vs Upper	0.947	0.462–1.941	0.882
Differentiation			**0.026**
Well	Ref‐	NA	NA
Moderate	1.500	0.995–2.261	**0.053**
Poor	2.167	1.207–3.891	**0.010**
Pathological type			
Squamous vs Adenocarcinoma	1.116	0.805–1.651	0.271
T stage			0.001
Tis/I	Ref‐	NA	NA
II	1.709	0.822–3.555	0.151
III	2.689	1.494–4.839	**0.001**
IV	3.543	1.614–7.779	**0.002**
N stage			
N0–N1 vs N2–N3	3.904	2.602–5.857	**<0.001**
BMI			
≥22 vs <22	0.752	0.527–1.075	0.118
NLR			
≥2.22 vs <2.21	1.438	1.004–2.060	**0.047**
PLR			
≥123.51 vs <123.51	1.552	1.083–2.225	**0.017**
AGR			
≥1.61 vs <1.61	0.490	0.341–0.705	**<0.001**
LDL (mmol/L)			
≥2.42 vs <2.42	0.754	0.527–1.079	0.122
PNI			
≥50 vs <50	0.563	0.394–0.806	**0.002**
CEA (ng/mL)			
≥5 µg/L vs <5 µg/L	2.038	1.259–3.299	**0.004**
PRL			
≥0.1 vs <0.1	3.974	2.758–5.726	**<0.001**

Multivariable analysis	HR	95% CI of HR	*P* value^a^
Age (years)			
≥65 vs <65	1.426	0.984–2.065	0.061
Sex			
Male vs female	2.417	1.123–5.204	0.024
N stage			
N0–N1 vs N2–N3	1.766	1.084–2.877	0.022
AGR			
≥1.61 vs <1.61	0.574	0.395–0.834	0.004
PNI			
≥50 vs <50	0.676	0.463–0.987	0.043
PRL			
≥0.1 vs <0.1	2.970	1.902–4.638	<0.001

Abbreviations: AGR, Albumin‐to‐globulin ratio; BMI, Body mass index; CI, confidence interval; HR, hazard ratio; LDL, Low‐density lipoprotein; NLR, Neutrophil‐to‐lymphocyte ratio; OS, Overall survival; PLR, Platelet‐to‐lymphocyte ratio; PNI, Prognostic Nutritional Index; PRL, Positive lymph nodes ratio.

Bold values are *P* values which less than 0.05 with statistic significance in both univariable and multivariable Cox regression.

^a^ Cox hazard regression analysis, *P* < 0.05 considered as statistical significance.

### 3.3 Visual nomogram establishment and internal/expanded validation

**FIGURE 2:**
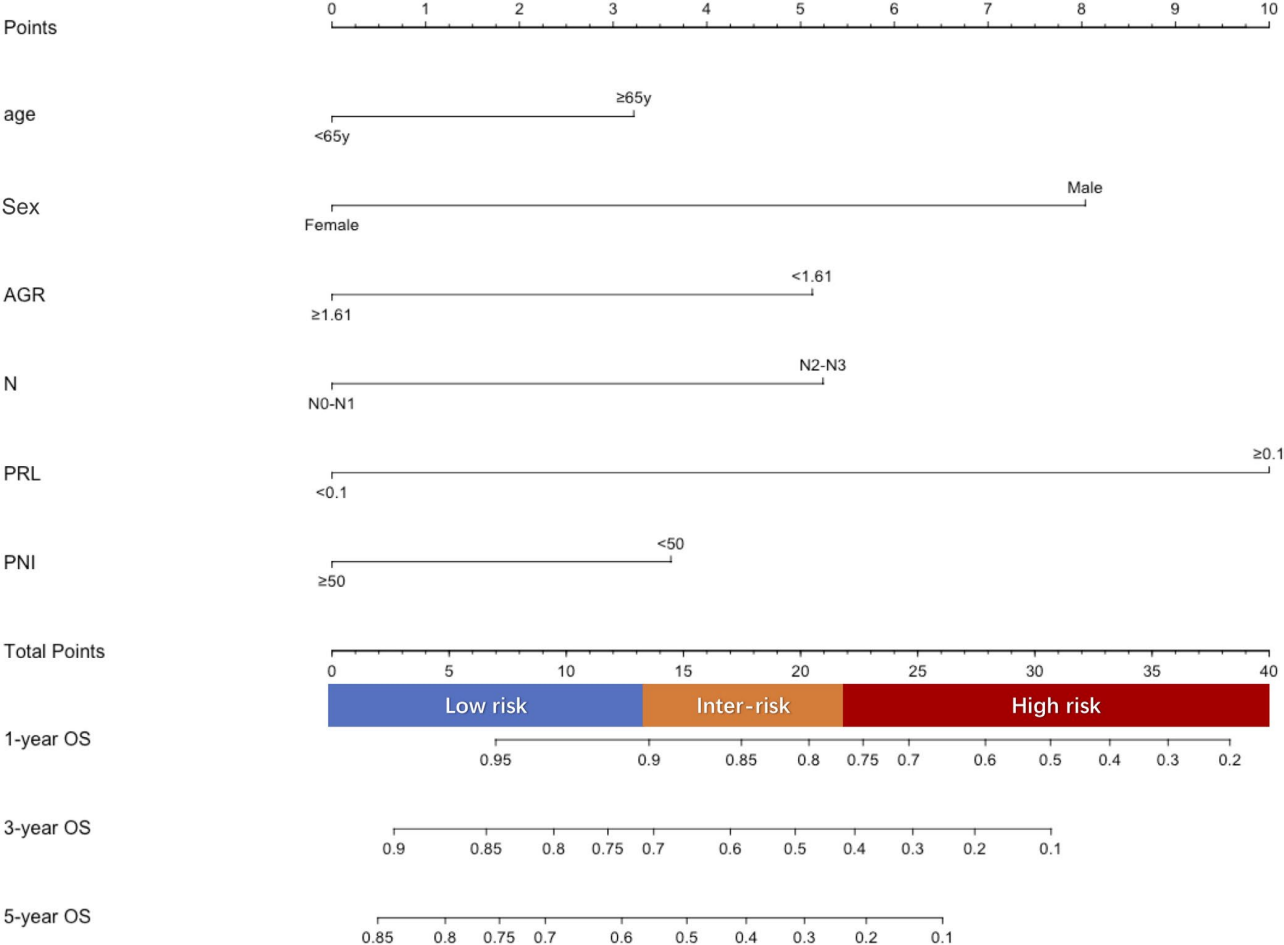
Nomogram prediction model for OS of EC patients underwent radical esophagectomy. As showed above, six variables correspond to the upper points scale, respectively, while the sum score of each variable reaches downward to the total points which is related to the prediction of 1‐, 3‐, 5‐year overall survival rate. Moreover, a line bar composed of three colors indicates three risk group according to the predictive OS. Furthermore, the risk status of radical esophagectomy EC patients would be obtained based on the nomogram. AGR, albumin‐to‐globulin ratio; PRL, positive lymph nodes ratio; PNI, Prognostic Nutritional Index; N, modified N staging; Inter risk, Intermediate risk

**FIGURE 3:**
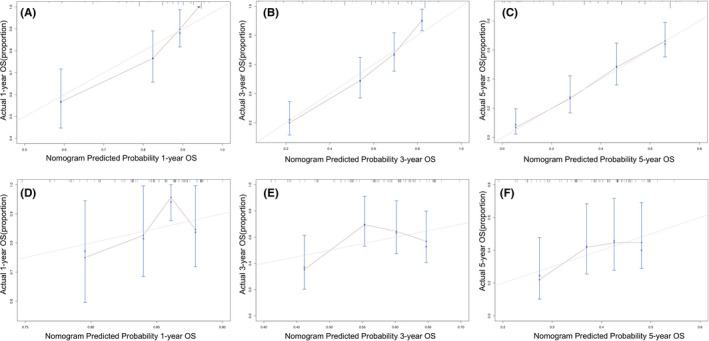
Calibration curves of nomogram model in predicting OS of EC patients. 1‐, 3‐, and 5‐year OS probability and actual 1‐, 3‐, and 5‐year OS in primary cohort (A–C). Calibration curves of nomogram model predicting 1‐, 3‐, and 5‐year OS probability and actual 1‐, 3‐, and 5‐year OS in validation cohort (D–F). The closer curve is to the calibration dotted line, the more accurate the prediction capacity of nomogram. OS, Overall survival

Based on six independent factors obtained in multivariable Cox hazard regression analysis, a nomogram characterized by scale line and score weight reflected to 1‐year, 3‐year, 5‐year OS prediction was established (Figure 2). The C‐index and calibration curve were used in the primary and validation cohorts for internal/expanded validation, respectively. The C‐index of the nomogram was 0.75 (95% CI 0.68–0.82) in the primary cohort and 0.70 (95% CI 0.65–0.75) in the validation cohort, which demonstrated its outstanding prediction accuracy. Furthermore, a set of calibration curves demonstrated the consistency between actual OS and nomogram‐predicted OS (Figure 3).

### 3.4 ROC of nomogram in prediction OS compared with AJCC T stage and N stage

**FIGURE 4:**
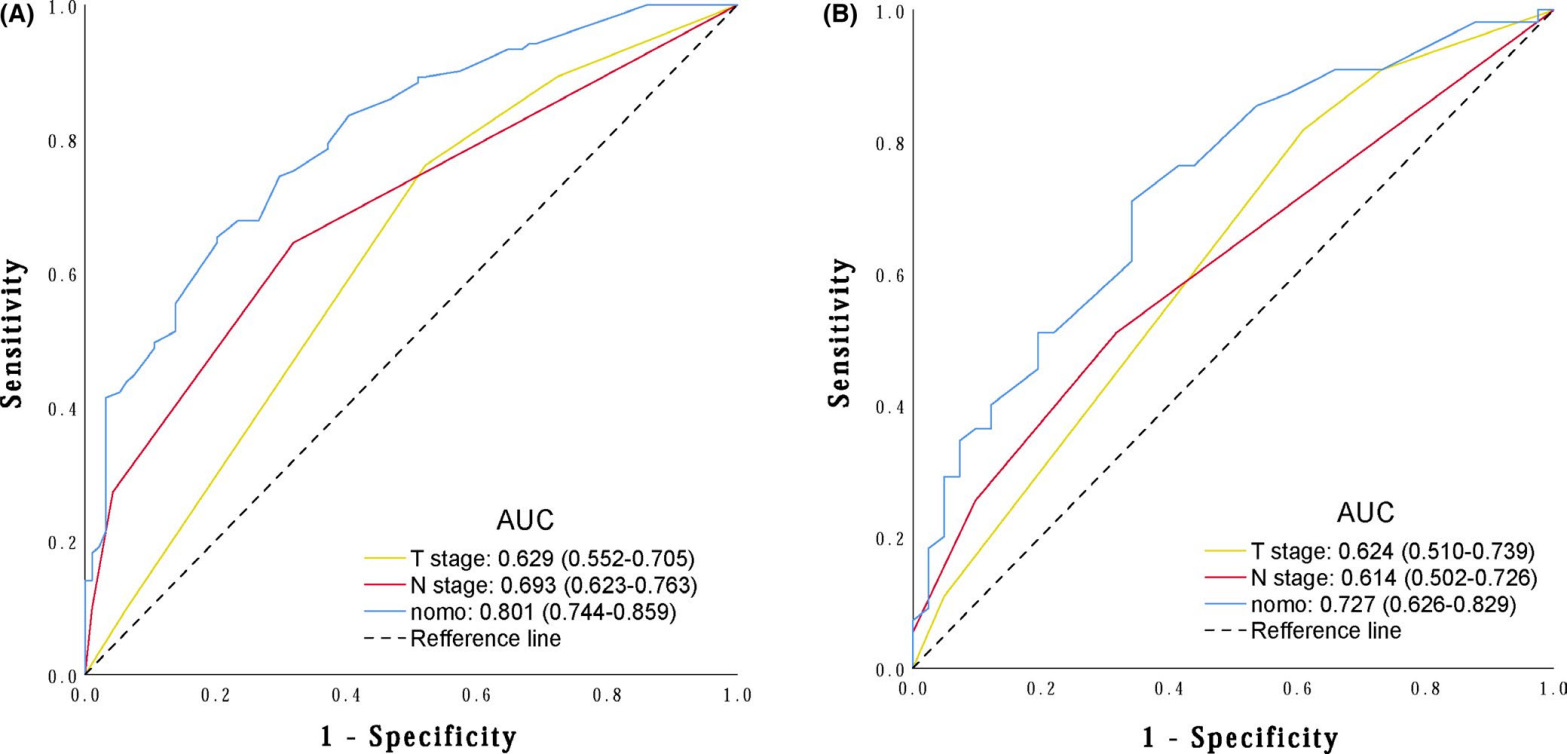
ROC curves of TNS calculated from nomogram model for the OS prediction were performed in both primary cohort (A) and validation cohort (B) compared with 7th AJCC T and N stages. The larger value of AUC reveals a better capability for OS prediction, ROC: receiver operating characteristics, TNS: total nomogram‐related score, AUC: area under curve, nomo: Nomogram

The total nomogram‐related score (TNS) was calculated for each patient as the summation of scores corresponding to the status of variables in nomogram. The ROC of TNS, conventional 7th AJCC T and N stages for OS prediction were plotted (Figure 4). The area under the curve (AUC) was clear in TNS (0.801; 95% CI 0.744–0.859) compared with T stage (0.629; 95% CI 0.552–0.705) and N stage (0.693; 95% CI 0.623–0.763) in the primary cohort (Figure 4A). In the validation cohort, the AUC of TNS was 0.727 (95% CI 0.626–0.829), which was distinct from that of the T stage (0.624; 95% CI 0.510–0.739) and N stage (0.614; 95% CI 0.502–0.726) (Figure 4B). This result indicates that TNS has better OS prediction capability for patients who underwent radical esophagectomy.

### 3.5 Nomogram‐based risk classification system

**FIGURE 5:**
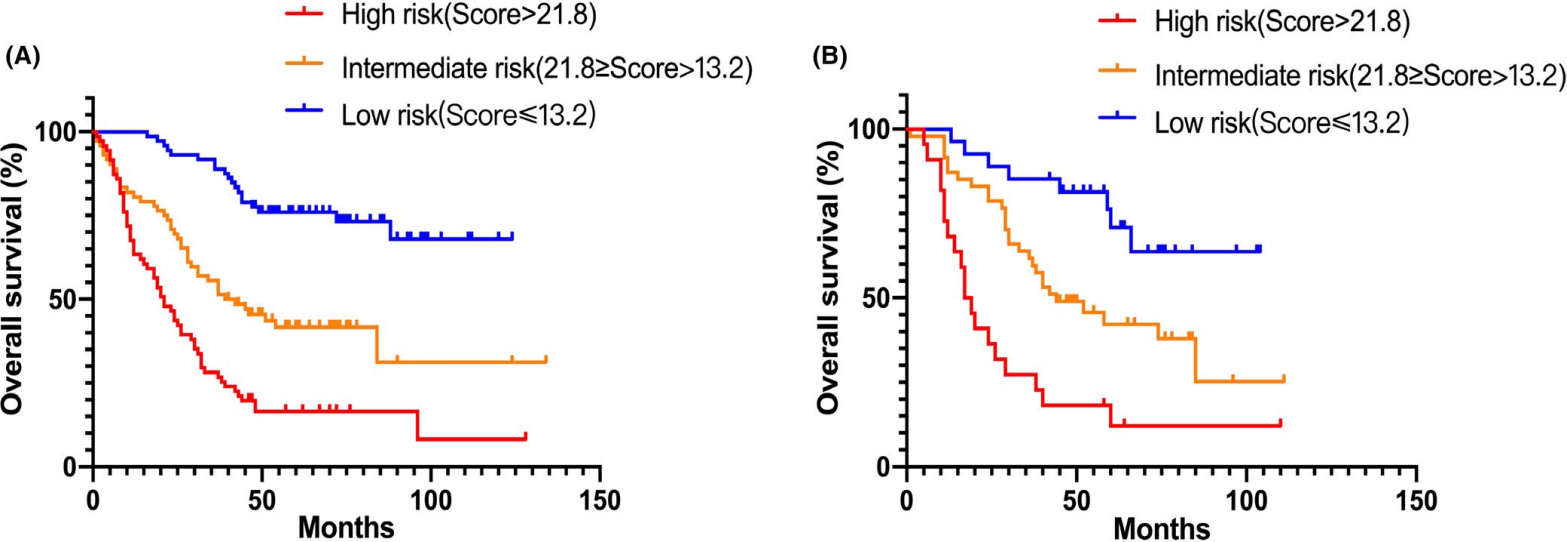
Kaplan–Meier curves for the OS of EC patients in the low‐, intermediate‐, and high‐risk groups of primary (A) and validation cohorts (B), respectively. The cut‐off scores were determined by TNS in primary cohort. OS, Overall survival; TNS, total nomogram‐related score

In the primary group, all patients were equally divided into high‐ (71/215, score ≥21.8), intermediate‐ (72/215, 21.8 > score ≥ 13.2), and low‐risk groups (72/215, score <13.2) according to TNS. The Kaplan–Meier curves showed significant difference in the primary cohorts, with a median OS of not applicable (NA), 51 months, and 18 months in low‐, intermediate‐, and high‐risk groups, respectively (Figure 5A). The hazard ratios (HRs) of intermediate‐ and high‐risk groups were 3.221 (95% CI 1.933–5.368; *P* < 0.001) and 6.329 (95% CI 3.964–10.11; *P* < 0.001), respectively, referring to low risk group. In addition, equivalent risk classification was applied in validation cohort (Figure 5B), and analogous results were found. The median OS was NA, 44 months, and 28 months in low‐, intermediate‐, and high‐risk groups, respectively, while the HRs of intermediate‐ and high‐risk groups were 2.684 (95% CI 1.688–5.188; *P* = 0.009) and 5.721 (95% CI 2.499–13.10; *P* < 0.001), respectively, referring to low‐risk group (Table 3).

**TABLE 3:** Log‐rank test of the risk groups based on the nomogram model in primary and validation cohorts

	Primary cohort			Validation cohort		
	Low	Inter‐	High	Low	Inter‐	High
No. (%)	72 (33.5)	72 (33.5)	71 (33.0)	38 (39.6)	41 (42.7)	17 (17.7)
Median OS (months)	NA^a^	51	18	NA^a^	44	28
HR	Ref‐	3.221	6.329	Ref‐	2.684	5.721
95%CI of HR	Ref‐	1.933–5.368	3.964–10.11	Ref‐	1.688–5.188	2.499–13.10
*P* value^b^	Ref‐	<0.001	<0.001	Ref‐	0.009	<0.001

Abbreviations: CI, confidence interval; HR, hazard ratio; Inter‐, Intermediate; OS, Overall survival.

^a^ Less than half of the deaths during the entire follow‐up.

^b^ Log‐rank test, *P* < 0.05 considered as statistical significance.

### 3.6 Comparison of nomogram‐related risk classification system with TNM staging

**FIGURE 6:**
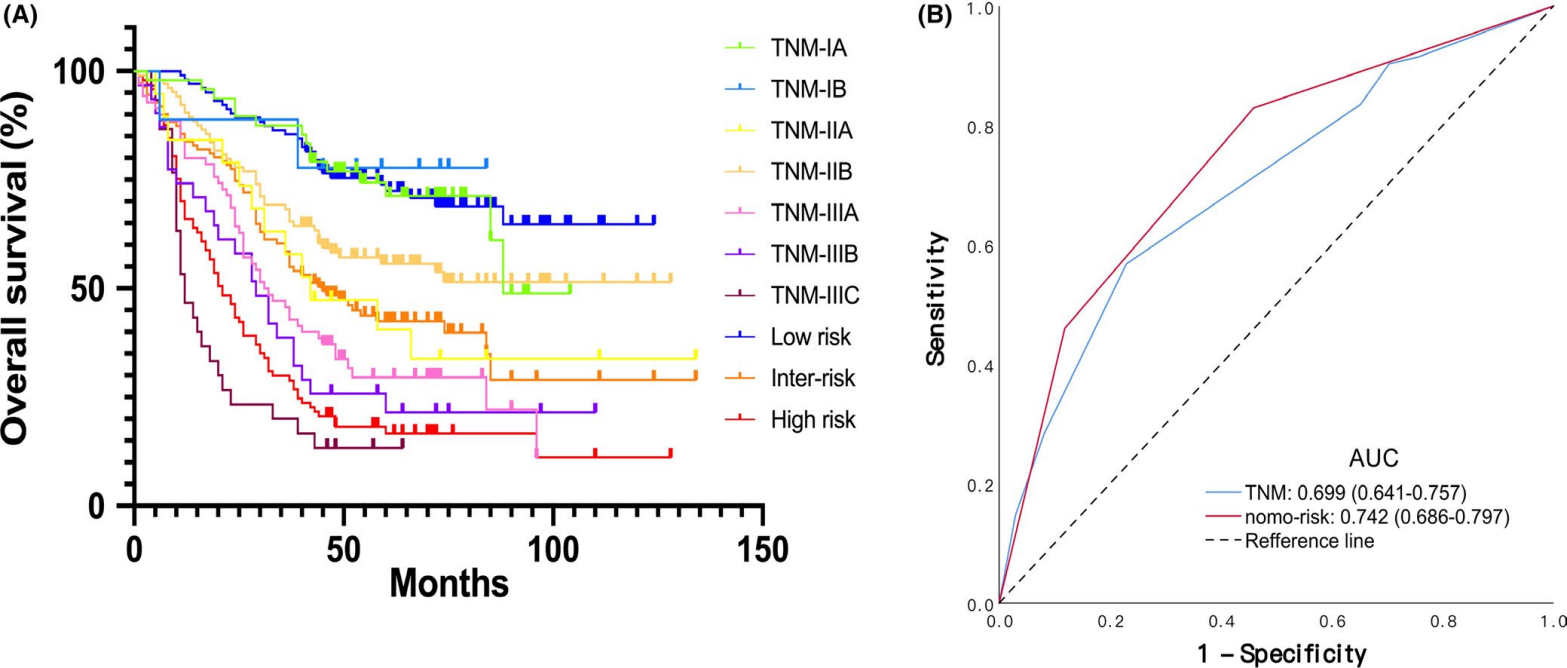
Kaplan–Meier curves for the OS of EC patients in AJCC TNM stages and nomogram‐related risk groups (A), ROC curves of AJCC TNM stages and nomogram‐related risk groups in predicting OS in EC patients (B). OS, Overall survival; nomo‐risk, nomogram‐related risk classification system; Inter‐, Intermediate; AJCC TNM, tumor–node–metastasis staging system represented by 7th American Joint Committee on Cancer

All patients in the total cohort were divided into different risk groups according to the calculated TNS. The R*C contingency in Table 4 shows that the risk classification was strongly correlated to TNM stages (*r* ^2^ = 0.647, *P* < 0.001). Additionally, Kaplan–Meier curves in Figure 6A showed significant differences in OS of patients between risk classification and TNM stages (log‐rank, *P* < 0.001), and larger AUC in risk classification system than TNM stages (0.742 vs 0.699) is displayed in Figure 6B.

**TABLE 4:** R*C contingency tables of all patients with TNS‐related risk in correlation with AJCC TNM stage.

	Low risk (N)	Inter risk(N)	High risk(N)	Total(N)	*P* value^b^	Correlation^c^
TNM stage						
IA^a^	30	18	0	48		
IB	6	3	0	9		
IIA	7	12	0	19		
IIB	49	45	10	104	<0.001	*r* ^2^ = 0.647
IIIA	10	26	34	70		*P* < 0.001
IIIB	1	6	24	31		
IIIC	0	1	29	30		
Total	103	111	97	311		

Abbreviations: AJCC, American joint Committee on cancer; Inter‐, Intermediate; TNS, Total nomogram‐related score.

^a^ According to the definition of 7th AJCC TNM staging system.

^b^ chi‐square test.

^c^ Pearson Correlation analysis, *P* < 0.05 considered as statistical significance.

## 4 DISCUSSION

The prognostic factors in EC patients are known to be complicated; thus, an accurate and comprehensive prognostic method is of great significance for the evaluation of EC and optimization of treatment strategies. To date, the TNM staging system proposed by AJCC has been widely used for the assessment of cancers, including EC. For patients who are adapted to surgery, accurate T and N staging is the most crucial task.^23^ However, the TNM staging system mainly represents anatomical relevance and sometimes does not fully reflect the prognosis. Therefore, recently, the use of nomograms, which can incorporate multiple prognostic factors, has been widely investigated to predict cancer‐related survival, recurrence, and metastasis with considerable potential.^24^, ^25^, ^26^, ^27^ OS is regarded as the most commonly used outcome considered to indicate cancer‐related survival prognosis with confounding factors, but there only few nomogram studies on EC‐related OS prediction. In this study, we included some classic and novel factors that are clinically available to establish a nomogram for prediction verification and clinical assessment, which is unprecedented and innovative.

Based on literature review and clinical experience, a set of factors were included in the initial analysis of EC patients who underwent radical esophagectomy. In addition to some conventional demographic and clinicopathological factors, a cluster of verified cancer‐related prognostic factors, including AGR, NLR, PLR, PRL, LDL, and PNI, may be used to establish nomograms.^28^, ^29^ Among these, six factors—age, sex, N stage (with modified), AGR, PRL, and PNI—were identified as independent prognostic factors by Cox hazard regression analysis, and the HR of each factor displays the weight of score in the nomogram.^9^ The accuracy of nomogram in predicting OS in the primary and validation cohorts was tested through the C‐index value and calibration curve, and considerable accuracy and consistency were obtained. To further investigate its clinical significance, we compared the nomogram with AJCC T and N staging system using ROC curve and AUC. In the aforementioned results, our nomogram presented a larger AUC value, which suggests more outstanding predictive ability.^30^ Subsequently, we calculated the TNS of each patient and three risk classification groups were formulated according to the level of score. The OS of the three groups showed a significant difference in both cohorts. Finally, we performed an analysis based on the risk classification system in the total cohort, comparing it with TNM staging to identify its clinical applicability. The risk classification system showed strong correlation with TNM staging (*r* ^2^ = 0.647, *P* < 0.001). Based on log‐rank test findings and ROC curve analysis, the nomogram exhibited better efficacy than TNM staging in terms of OS prediction.

In terms of demographic characteristics, we found that two factors—age and sex—were associated with the OS of EC patients after radical esophagectomy, and thus, these factors were included in the establishment of our novel nomogram. Based on the findings of previous and current studies, men have higher incidence and mortality rate than women in various malignancies.^1^, ^31^, ^32^ In addition to cancer‐related factors, it cannot be ignored in the prognosis of elderly cancer patients because the pathological mechanisms induced by aging may cause more nutritional and metabolic diseases and disorders, such as amyotrophy, metabolism damage, and neurological disease, contributing to the impediment of longevity.^33^ Our study results showed that age >65 years was an independent factor for OS prediction, conforming to the interpretation mentioned above.

Furthermore, in comparison with some nomogram studies that assessed resectable EC patients, Shao's study focuses on the predictive ability of inflammation‐related factors for OS, and accordingly builds a nomogram model. His study is partially similar to our study, and some factors used in his study, including PLR and NLR, were selected to be used in our initial analysis.^34^ However, with no statistical difference found, they were removed from the multivariable Cox hazard regression analysis in our study. This indicates that the factors affecting the OS of patients with radically resected EC are very complicated. PLR and NLR are not only related to the prognosis of malignant tumors, but also related to inflammatory diseases, such as rheumatic diseases and cardiovascular diseases.^35^, ^36^ EC is dependent on the complex interaction between the tumor and the hosts' inflammatory response, so the distinction between PLR and NLR shown in Shao's study and ours implies that the independence of factors affecting prognosis is unseparated from the integrity of the individual physiology.^37^ As mentioned before, a low AGR is associated with increased cancer mortality. A study assessing generally healthy individuals proved that AGR is a risk factor for cancer incidence and mortality in both short‐ and long‐term cohorts.^38^ There is biological plausibility for the link between low AGR and increased cancer incidence and mortality: an increase in cytokines in the tumor microenvironment may elevate the total protein levels, with induced albumin synthesis suppression in the liver.^39^, ^40^ In patients with EC, malnutrition, relevant to decreased serum albumin and AGR levels, could be another mechanism of poor survival. PNI is composed of serum albumin level and lymphocyte count as a calculative index, which is widely utilized in prognostic evaluation of various malignancies. As a factor reflecting tumor‐related nutritional status and system inflammation response, the role of PNI in EC patients' prognosis is highly desirable. Kazuo's study demonstrated the predictive role of PNI in EC patient outcomes and its inseparable relationship with tumor‐infiltrating lymphocytes (TILs).^17^ PNI was proven to be significantly associated with OS in Cox hazard regression analysis, with a negative correlation of HR of 0.676 (95% CI 0.463–0.987) in our study, and it was first included in nomogram for OS prediction in resectable EC patients. Moreover, the laboratory‐sourced factors that we selected were all preoperative to eliminate the inflammatory and metabolic bias caused by surgery.

For patients with resectable EC, although surgeons have different skills, the degree of lymph node dissection is considered an effective adjudication, and extended lymph node dissection significantly improves the prognosis of EC patients.^41^, ^42^ Recently, the ratio of positive retrieved lymph nodes to total number of retrieved lymph nodes, PRL, has been shown to be a superior indicator of survival of EC patients.^43^, ^44^ Compared with the N staging of AJCC, it not only reflects the quantity but also the extent of the lymphatic metastasis, especially for EC patients with less than two‐field lymph node dissection or underestimated N staging due to insufficient retrieval of positive lymph nodes.^45^ Evidently, PRL showed a higher HR of 2.970 (95% CI 1.902–4.638) and the greatest weight in nomogram compared with N staging in our study.

A number of studies have assessed the superiority and clinical significance of nomograms in comparison to AJCC staging.^10^, ^18^ In our study, compared with T and N staging, we observed that the AUC of nomogram in the primary cohort reached 0.801 (95% CI 0.744–0.859), which was significantly higher than that of the T and N staging. Despite the fact that the AUC of nomogram in validation cohort dropped to 0.727 (95% CI 0.626–0.829), it is still superior to T and N staging in predicting OS. Moreover, to verify the clinical significance of the proposed risk classification system, we utilized TNM staging as a reference uncommonly, highlighting the clinical significance and the value of the risk classification system in OS prediction in comparison with TNM staging.

Additionally, in contrast with studies on nomogram in EC patients, PNI, which is scarcely investigated in nomograms of EC patients, was integrated into our study, together with internal and expanded validation. In addition, an applicable risk classification system based on nomogram was developed in our study.^13^, ^18^, ^34^ Then, we compared this nomogram with the AJCC staging comprehensively, which showed preferable prognostic value in this nomogram.

Several limitations exist in our study. First, it was a retrospective analysis with probably inherent bias. Second, the primary and validation cohorts of this study were from a single center. Third, the threshold index of the factors mentioned in our study is heterogeneous. Therefore, a large‐sample, multi‐center, multi‐parameter research needs to be further verified.

## 5 CONCLUSIONS

We established and verified a nomogram composed of six clinically accessible indicators, including a corresponding risk classification system for OS prediction in EC patients who underwent radical esophagectomy. The nomogram is novel and practical, showing considerable accuracy and efficacy compared with the conventional AJCC staging system. Our results could be used in promising clinical applications prospectively, such as patient counseling, convenient prognosis assessment, and individualized follow‐up strategy formulation, promoting the combination of prognostic tools and clinical management for operable EC patients.

## 6 ETHICS APPROVAL

This study was conducted in accordance with the principles of the Declaration of Helsinki, all participating patients signed an informed consent form and this study was approved by the Clinical Research Ethics Committee of The First Affiliated Hospital of Zhejiang University School of Medicine.

## CONFLICTS OF INTEREST

The author declares that there is no conflict of interest that could be perceived as prejudicing the impartiality of the research reported.

## AUTHOR CONTRIBUTIONS

All the authors contributed to the completion of this manuscript, including the design of study (Xinye Li and Jian Hu), collection of clinical data (Jinming Xu, Sijia Yang, and Li Yu), statistical analysis (Xinye Li and Linhai Zhu), draft writing (Xinye Li and Wang Lv), and study guidance and assistance (Jian Hu).

## Data Availability

Some or all data generated or used during the study are available from the corresponding author by request.
